# The Combined Effects of Citric Acid and Ammonium Nitrate on the Phytoremediation of Cd-Phenanthrene Co-Contaminated Soil Using Marigold

**DOI:** 10.3390/toxics14090770

**Published:** 2026-08-28

**Authors:** Xinzhuo Qian, Ting Xu, Xuemei Zhong, Weijin Zheng, Lizhu Yuan, Bo Song

**Affiliations:** 1 College of Earth Sciences, Guilin University of Technology, Guilin 541004, China; 2 College of Environmental Science and Engineering, Guilin University of Technology, Guilin 541004, China; 3 Guangxi Kangwanjia Farmland Soil Remediation Co., Nanning 530006, China; 4 Research Center for Eco-Environmental Engineering, Dongguan University of Technology, Dongguan 523808, China

**Keywords:** co-contamination soil, citric acid, nitrogen fertilizer, phytoremediation

## Abstract

Citric acid (CA) and nitrogen (N) fertilizers have been widely applied to enhance phytoremediation of various contaminated soils, yet their combined effects and underlying mechanisms in cadmium (Cd)–polycyclic aromatic hydrocarbon (PAH) co-contaminated systems remain poorly understood. Here, we investigated the combined effects of CA and ammonium nitrate on the phytoremediation of Cd–phenanthrene (Phe) co-contaminated soil using marigold (*Tagetes patula* L.), with random forest, mantel tests, and structural equation modeling employed to explore hypothesized pathways and associations. The results demonstrated that combined CA and N application significantly promoted plant growth, Cd uptake, and Phe dissipation. Among the tested treatments, the low-dose combination (1 g CA + 0.1 g N pot^−1^) increased plant biomass by 88%, enhanced shoot Cd uptake by 121%, and achieved a Phe dissipation rate of 76.56%, representing the optimal remediation strategy. Mechanistically, the synergy between CA and N extends beyond simple growth promotion—N drives biomass production and, independently, facilitates Cd mobilization through rhizosphere acidification, while CA enhances contaminant bioavailability and buffers N-induced salt stress, together maintaining rhizosphere ionic homeostasis for efficient Cd translocation and Phe dissipation. This study provides a mechanistic framework for designing combined amendment strategies to enhance phytoremediation of co-contaminated soils.

## 1. Introduction

The co-contamination of agricultural soils with heavy metals and organic pollutants represents a pressing global environmental challenge, posing well-documented threats to both ecosystem integrity and food safety [1,2]. Cadmium (Cd) is among the most hazardous heavy metals owing to its pronounced toxicity, environmental mobility, and tendency to accumulate through trophic chains [3,4]. Polycyclic aromatic hydrocarbons (PAHs), such as phenanthrene (Phe), constitute another major category of persistent organic contaminants, with origins ranging from fossil fuel combustion to various industrial processes [5]. The coexistence of Cd and PAHs in soils is frequently observed at industrial sites, e-waste recycling areas, and agricultural lands irrigated with contaminated water [6,7], and often produces synergistic toxic effects on soil biota and plants that complicate remediation and heighten ecological risks [7]. Phytoremediation has been increasingly recognised as a cost-effective and environmentally benign alternative for managing such co-contaminated soils, drawing on the intrinsic capacity of plants to extract, stabilise, or degrade pollutants [8,9]. However, the efficiency of phytoremediation is frequently constrained by two interrelated challenges: the low bioavailability of contaminants in the rhizosphere and the limited biomass production of many accumulator plants [10,11].

To overcome these bottlenecks, various chemical amendments have been explored to enhance pollutant mobilization and plant performance [12,13]. Among these, low-molecular-weight organic acids (LMWOAs), particularly citric acid (CA), have received considerable attention due to their biodegradability and their demonstrated potential to enhance both metal solubility and organic pollutant desorption from soil matrices [12,14]. For instance, exogenous CA application at 1 mmol·kg^−1^ significantly increased the aboveground Cd accumulation of Hydrangea macrophylla by 262.22% [12], while CA treatment at 5 mmol·kg^−1^ elevated the aboveground Cd uptake of Celosia argentea by 157% in naturally contaminated soils [15]. Similarly, nitrogen (N) fertilization has been widely reported to promote plant growth under stress conditions and to enhance the removal of co-contaminants [13,16]. In alfalfa-based phytoremediation systems, N addition elevated pyrene dissipation rates from 78.10% to 87.25% in PAH-Cd co-contaminated soil [17]. However, the effectiveness of both amendments is often constrained by dose-dependent trade-offs—excessive CA may induce phytotoxicity [8], while high N inputs can elevate soil electrical conductivity and impose osmotic stress on plants [18]. These limitations suggest that the combined application of CA and N at appropriate doses might circumvent the adverse effects of each amendment while harnessing their respective benefits. Nevertheless, the mechanisms underlying their potential synergy—particularly regarding the simultaneous promotion of plant growth, Cd phytoextraction, and Phe dissipation—remain insufficiently characterized.

In this study, we investigated the combined effects of CA and ammonium nitrate (NH_4_NO_3_) on the phytoremediation of Cd–Phe co-contaminated soil using marigold (*Tagetes patula* L.), a fast-growing ornamental plant with demonstrated remediation potential [19]. The specific objectives were to (1) evaluate the effects of different CA and N application rates, individually and in combination, on marigold growth, photosynthetic pigments, Cd accumulation, and Phe dissipation, and (2) elucidate the rhizosphere chemical mechanisms underlying CA-N synergy, with particular emphasis on their interactive regulation of soil pH, EC, and nutrient availability. The findings of this work are expected to provide a mechanistic framework and practical guidance for the design of effective phytoremediation strategies in co-contaminated soils.

## 2. Materials and Methods

### 2.1. Chemicals and Soil

The certified reference material for Cd (NO. GBW(E)085377, 1000 μg·mL^−1^) was purchased from the National Reference Material Center. Phe, along with organic solvents, concentrated acids, and inorganic salts, was sourced commercially from Sinopharm Chemical Reagent Co., Ltd. (Shanghai, China). Guaranteed-grade nitric and perchloric acids were utilized for digestion, while all other reagents met analytical grade specifications. Ultrapure water (18.2 MΩ·cm), produced via a Milli-Q IQ 7000 system (Millipore, Molsheim, France), was employed for preparing all working solutions. Seeds of marigold were provided by Suqian Sunrise Seed Industry Co., Ltd. (Suqian, China).

Surface agricultural soil, corresponding to a depth of 10–20 cm, was collected from a cultivated field located in Shenyang, China (123.606776° E, 41.918325° N). The background Cd level in this pristine soil was determined to be 0.10 mg·kg^−1^, with the remaining physicochemical characteristics detailed in our earlier investigation [20]. To generate the co-contaminated test matrix, the clean soil was artificially spiked with Cd(II) nitrate and Phe (CAS: 85-01-8, >97% purity), strictly adhering to the preparation protocol established in reference [20]. Following equilibration, the final concentrations of total Cd, available Cd, and Phe in the spiked soil were recorded as 2.85, 1.51, and 183.72 mg·kg^−1^, respectively. The Cd concentration was selected based on the risk intervention range (1.5–4.0 mg·kg^−1^) stipulated by the Chinese Soil Environmental Quality Standard (GB 15618-2018) [21] for agricultural soils, and is consistent with levels reported in contaminated field soils. The Phe concentration (183.72 mg·kg^−1^) is within the range commonly used in phytoremediation studies [22,23]. These values represent the initial contaminant concentrations at the start of the experiment.

### 2.2. Experiment Setup

The greenhouse-based pot trial was conducted under controlled environmental conditions. Illumination was provided by fluorescent lamps delivering a photosynthetic photon flux density of 54 μmol·m^−2^·s^−1^, with the photoperiod regulated via an automatic timer to maintain a 16-h light and 8-h dark cycle. The ambient temperature was thermostatically controlled at 24–26 °C during daylight hours and 18–20 °C at night. Prior to sowing, marigold seeds were surface-sterilized in a 2% (*v*/*v*) hydrogen peroxide solution for 30 min, followed by three rinses with ultrapure water, and subsequently germinated in uncontaminated soil. Upon reaching the six-leaf stage, healthy and uniform seedlings were selected and transplanted into plastic pots (12 cm in height × 13 cm in diameter), each filled with 0.8 kg of the artificially co-contaminated substrate, at a density of seven plants per pot. Throughout the experimental period, pots were irrigated on alternate days, and soil moisture was adjusted to approximately 60% of field water-holding capacity, which was routinely monitored by gravimetric determination through periodic weighing. The pots were randomly positioned in the greenhouse at the start of the experiment and periodically rotated every 7 days. After a 30-day acclimatization period following transplantation, chemical additives were introduced. Nine treatment combinations were established as outlined in Table 1. The applied additives consisted of citric acid and/or ammonium nitrate mixed solutions. The additives were applied on days 30, 37, and 44 after transplantation. The detailed application concentrations and volumes for each treatment are provided in Table S1. The solution was gently and evenly poured onto the soil surface around the plant stem to allow gradual infiltration. To ensure volumetric consistency across all treatments, the final volume of each application was equalized to 66.7 mL using ultrapure water. Each treatment was replicated three times. Notably, apart from the designated chemical supplements, no additional fertilization was applied to any of the pots during the entire greenhouse cultivation phase.

At the conclusion of the 30-day treatment period, leaf tissues were harvested for the quantification of chlorophyll and anthocyanin contents. Subsequently, both soil and plant samples were collected. All seven plants from each pot were collected and pooled as a single sample. The harvested plants were separated into shoot and root fractions. The root portions were thoroughly washed with running tap water, followed by ultrapure water, and then immersed in a 20 mM Na_2_EDTA (ethylenediaminetetraacetic acid disodium salt) solution for 15 min to eliminate surface-bound metal ions, after which they were rinsed three additional times with ultrapure water. All cleaned plant materials were initially oven-dried at 105 °C for 20 min to inactivate enzymatic activity, and subsequently dehydrated at 70 °C until a constant dry weight was achieved. The final biomass was recorded using a calibrated analytical balance.

### 2.3. Analytical Method

Leaf chlorophyll (Chl) and anthocyanin (Anth) concentrations were non-destructively assessed between 09:00 and 11:00 a.m. using a Dualex Scientific optical leaf-clip device (Force-A, Paris, France). The pH and electrical conductivity (EC) of the soil matrix were determined from slurries prepared at a 1:2.5 (*w*/*v*) soil-to-water ratio, employing a S220 Seven Compact pH meter (Mettler-Toledo, Greifensee, Switzerland) and a DDSJ-308A conductivity meter (INESA, Shanghai, China), respectively. For nitrogen fractions, nitrate nitrogen (NO_3_^−^_N) was extracted with 0.01 mol·L^−1^ CaCl_2_ and subsequently quantified via UV–visible spectrophotometry, whereas ammonium nitrogen (NH_4_^+^_N) was isolated using 1 M KCl and measured by the indophenol blue colorimetric method. Available phosphorus (AP) was extracted with 0.5 mol·L^−1^ NaHCO_3_ and colorimetrically assayed through the molybdenum blue procedure. Available potassium (AK) was leached with 1.0 mol·L^−1^ CH_3_COONH_3_ and determined by atomic absorption spectrophotometry [24]. Bioavailable cadmium (Cd) was extracted with a DTPA-complexing solution containing 5 mmol·L^−1^ diethylenetriaminepentaacetic acid, 10 mmol·L^−1^ triethanolamine, and 10 mmol·L^−1^ CaCl_2_ (pH 7.3), following the protocol described in reference [24]. For total Cd quantification in marigold tissues, dried samples were digested in a mixture of HNO_3_ and HClO_4_ at 180 ± 5 °C for 120 min. The resulting digestates were passed through 0.22 μm membrane filters and analyzed by inductively coupled plasma mass spectrometry (ICP-MS, iCAP RQ, Thermo Fisher Scientific, Waltham, MA, USA). The instrument was calibrated using a five-point standard curve, yielding a correlation coefficient of R^2^ = 0.999. Procedural blanks were processed in parallel with each digestion batch; the blank average concentration was determined to be 0.013 μg·L^−1^. The limits of detection (LODs) and quantification (LOQs) were 0.005 μg·L^−1^ and 0.041 μg·L^−1^, respectively. Method accuracy was validated using plant standard reference material (GBW07602), which was analyzed in triplicate for each digestion batch. The relative standard deviation (RSD) for the certified reference material measurements was 0.023, and the recoveries ranged from 84.2% to 116.7% across all batches.

Residual Phe in soil was serially extracted three times with a dichloromethane/acetone (1:1, *v*/*v*) mixture, each cycle comprising 30 min of orbital shaking followed by 30 min of ultrasonication. The combined supernatants were concentrated under reduced pressure and redissolved in 2.0 mL of methanol. After filtration through 0.22 μm syringe filters, the extracts were injected (1 μL) into a gas chromatography–mass spectrometry system (GC-MS, ISQ QD 300, Thermo Fisher Scientific), equipped with a DB-5MS capillary column (30 m × 0.25 mm i.d. × 0.25 μm film thickness). Helium (99.999% purity) served as the carrier gas at a constant flow rate of 1.0 mL·min^−1^. The oven temperature program commenced at 80 °C, ramped at 20 °C·min^−1^ to 300 °C, and maintained for 10 min. The splitless injector was held at 280 °C, while mass spectrometric detection was performed in electron impact (EI) mode at 70 eV, with full-scan acquisition across the *m*/*z* 30–220 range at a scan rate of 0.3 s·scan^−1^. The instrument was calibrated using a five-point standard curve, yielding a correlation coefficient of R^2^ = 0.996. Procedural blanks were processed in parallel with each extraction batch, and all blank values were below the limit of detection. The limit of detection (LOD) was 1.231 μg·L^−1^. The limit of quantification (LOQ) was estimated as 3.693 μg·L^−1^. Method accuracy was validated through spike recovery experiments, in which clean soil was spiked with Phe at a concentration of 50 mg·kg^−1^ and extracted following the same procedure. Three replicate extractions were performed, yielding recoveries ranging from 81.7% to 108.4%, with a standard deviation of 7.56.

### 2.4. Statistical Analyses

The bioconcentration factor (BCF), translocation factor (TF), and cadmium uptake amount (Up) for root and shoot tissues were derived from the following equations:

BCF = Cp/Cs(1)

TF = Cp/Cr(2)

Up = Cp × Mp(3)

where Cp and Cr represent the Cd concentration (mg⋅kg^−1^) in the aboveground portion and roots, respectively. Cs denotes the Cd concentration in soil (mg⋅kg^−1^); Mp stands for the dry biomass of the shoot per pot (kg per pot).

All experimental results are expressed as arithmetic means with standard deviations (SDs). One-way analysis of variance (ANOVA) and two-way factorial analysis of variance (ANOVA) were employed to test for significant differences among treatment groups, using SPSS (IBM SPSS Statistics 22, New York, NY, USA) and a significance threshold of *p* < 0.05. Graphical representations of the measured variables were prepared with Origin software (version 8.0, OriginLab Corporation, Northampton, MA, USA). For the multivariate assessments, random forest (RF) analysis was performed using the randomForest package in R (version 4.3.1) to identify the most important predictors of Cd accumulation and Phe dissipation. Full RF parameters, including ntree, mtry, OOB metrics, cross-validation results, and variable importance rankings, are provided in Table S2. Mantel tests were performed using the mantel_test function from the linkET package in R (version 4.3.1) to examine correlations between soil/plant factor matrices and response variables (Cd uptake and Phe dissipation). Euclidean distance matrices were computed for both predictor and response variables using the vegdist function (vegan package) with the ‘euclidean’ method. Significance was assessed using 9999 permutations with a two-sided alternative hypothesis. To account for multiple comparisons across variable pairs, *p*-values were adjusted using the false discovery rate (FDR) method (Benjamini–Hochberg). And structural equation modeling (SEM) was adopted to examine hypothesized relationships among variables.

## 3. Results

### 3.1. Plant Biomass and Leaf Pigment Responses

**Figure 1:**
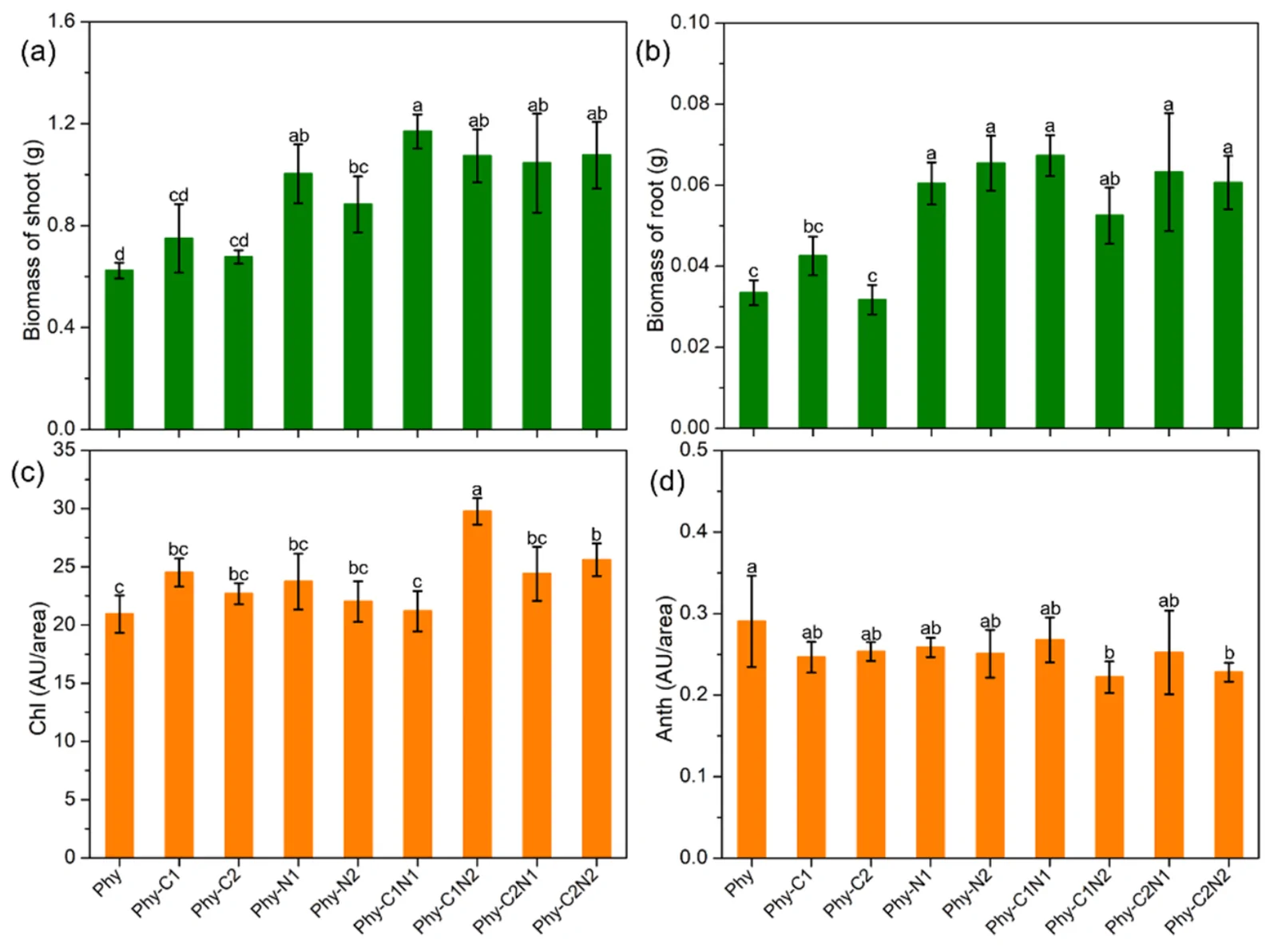
Effect of CA and N levels on the biomass and leaf pigment responses of Marigold: (**a**) biomass of shoots, (**b**) biomass of roots, (**c**) Chl, and (**d**) Anth. Each value represents the mean ± standard deviation of three independent experiments. Different lowercase letters on the column represent significant differences in different treatments in CA and N levels (*p* < 0.05).

The results show that for the main measured parameters, the CA × N interaction was not significant (all *p* > 0.05) (Table S3). The shoot and root biomasses of marigold under the different treatments are presented in Figure 1a,b. In the control (Phy) treatment, the shoot and root dry weights were 0.623 g and 0.033 g, respectively. Compared with the Phy control, the addition of CA alone did not cause statistically significant changes in either shoot or root biomass. In contrast, all treatments containing N significantly enhanced both shoot and root biomass. Among the combined applications, the low-concentration CA plus N treatment (Phy-C1N1) yielded the maximum biomass values, with shoot and root dry weights reaching 1.170 g and 0.067 g, which were 1.88-fold and 2.01-fold those of the Phy treatment, respectively. Increasing the concentration of either CA or N led to slight reductions in biomass, but these differences were not significant relative to the Phy-C1N1 treatment.

Regarding leaf Chl content (Figure 1c), slight increases were observed in the CA-alone, N-alone, Phy-C1N1, and Phy-C2N1 treatments, although none of these changes were statistically significant. However, the Phy-C1N2 and Phy-C2N2 treatments exhibited significant Chl enhancement, with the highest value recorded in the Phy-C1N2 treatment. The Anth content showed an opposite trend: modest, non-significant decreases occurred in the CA-alone, N-alone, Phy-C1N1, and Phy-C2N1 groups, while the Phy-C1N2 and Phy-C2N2 treatments displayed significant reductions in anth levels.

### 3.2. Cd Accumulation in Plant Tissues

**Figure 2:**
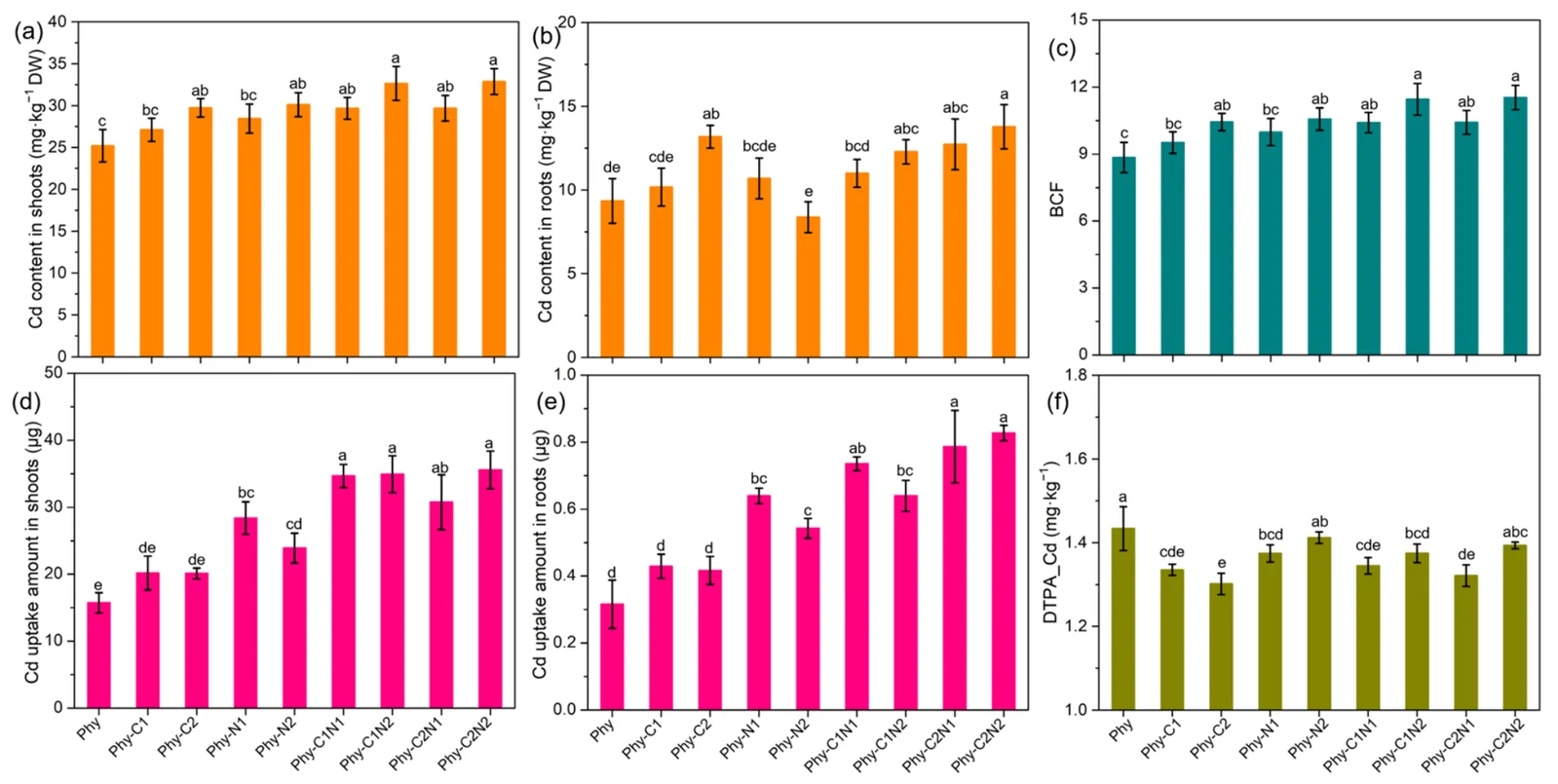
Effect of CA and N levels on Cd concentration, and BCF, TF and Cd uptake amounts of different plants: (**a**) Cd concentration in shoots, (**b**) Cd concentration in roots, (**c**) BCF, (**d**) Cd uptake amount in shoots, (**e**) Cd uptake amount in roots, (**f**) DTPA Cd concentration in soil. BCF refers to the bioconcentration factors of Cd. Each value represents the mean ± standard deviation of three independent experiments. Different lowercase letters on the column represent significant differences in different treatments in CA and N levels (*p* < 0.05).

The application of CA and N significantly influenced Cd concentrations in marigold plants (Figure 2). Relative to the Phy control, the low-concentration CA (Phy-C1) and N (Phy-N1) treatments did not cause notable changes in shoot Cd concentrations. In contrast, the addition of high-concentration CA or N, as well as their combined applications, significantly elevated shoot Cd levels. The highest shoot Cd concentration (32.86 mg·kg^−1^) was observed in the Phy-C2N2 treatment, representing a 30.4% increase over the control. For root tissues, no significant alterations in Cd concentrations were detected under the low CA, N alone, or Phy-C1N1 treatments. However, root Cd concentrations were markedly enhanced in the high-CA treatments (Phy-C2, Phy-C2N1, and Phy-C2N2) and in the Phy-C1N2 treatment. The bioconcentration factor (BCF) exhibited a response pattern analogous to that of shoot Cd concentration, with the maximum BCF value (11.53) recorded in the Phy C2N2 treatment.

Regarding Cd uptake amounts, the control treatment yielded a shoot uptake of 15.717 μg·pot^−1^. While sole CA addition did not result in a significant increase in shoot Cd uptake, all N-containing treatments (N alone or combined with CA) led to substantial enhancements. The highest shoot Cd uptake (35.571 μg·pot^−1^) was attained in the Phy C2N2 treatment, corresponding to 2.26-fold that of the control. A similar trend was observed for root Cd uptake, with the maximum value (0.827 μg·pot^−1^) occurring in the same treatment, which was 2.62-fold that of the control. Initially, the bioavailable Cd (DTPA-Cd) concentration in the co-contaminated soil prior to phytoremediation was 1.51 mg·kg^−1^. Following the various treatments, bioavailable Cd levels declined across all treatments. Compared with the Phy treatment, the high-N (Phy N2) and Phy C2N2 treatments did not exhibit significant changes in bioavailable Cd concentration, whereas all other treatments showed significant reductions.

### 3.3. Residual Phe in Soil

**Figure 3:**
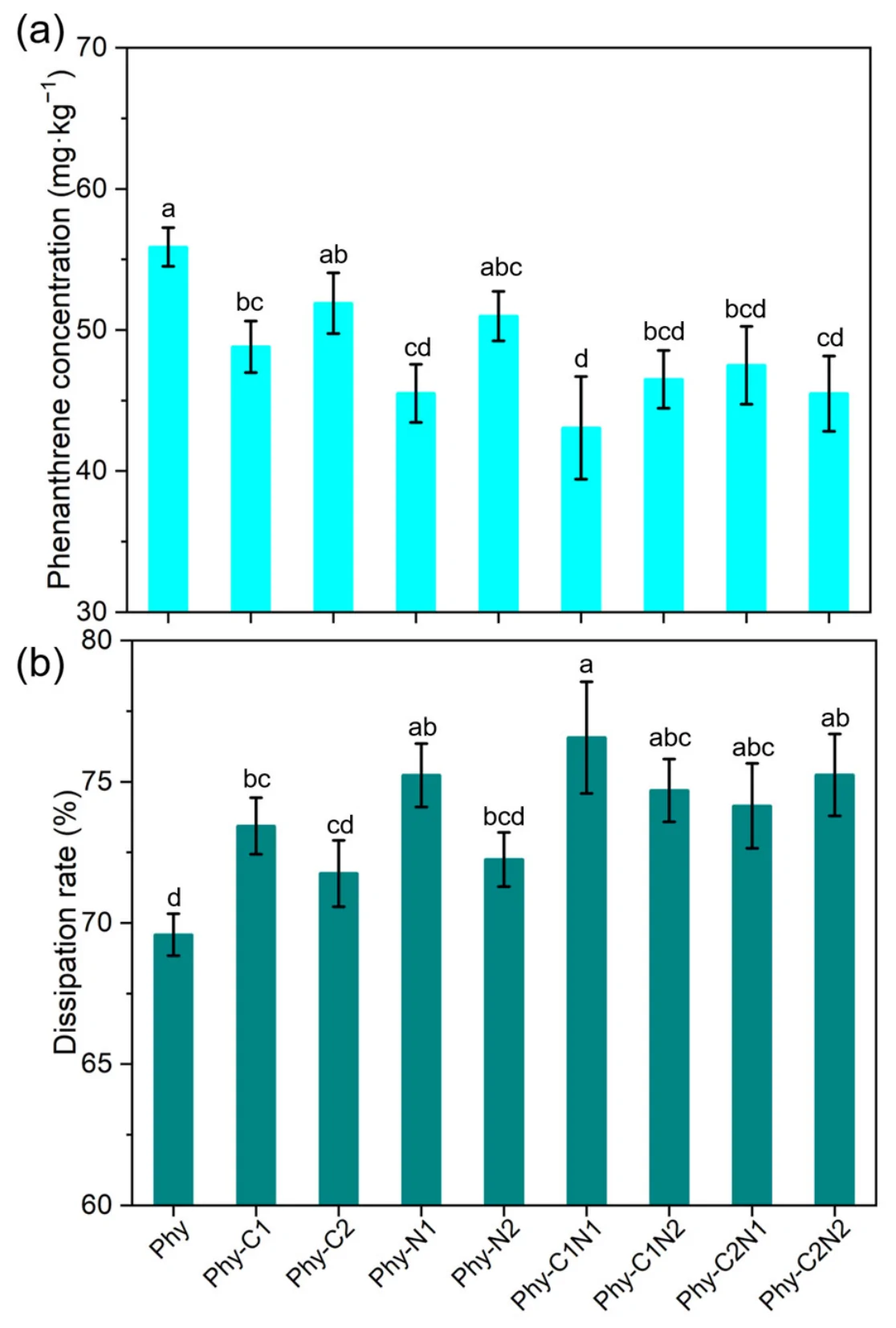
The residual concentration and dissipation rate of Phe in soil after different treatments. (**a**) Residual concentration of Phe; (**b**) dissipation rate of Phe. Each value represents the mean ± standard deviation of three independent experiments. Different lowercase letters on the column represent significant differences in different treatments in C14 alkane concentration (*p* < 0.05).

Prior to remediation, the initial Phe concentration in the co-contaminated soil was determined to be 183.72 mg·kg^−1^. Following the combined application of marigold and various chemical additives, residual Phe concentrations in the soil generally decreased relative to the control, with the extents of reduction varying across treatments (Figure 3). In the marigold-only control (Phy), the residual Phe concentration was 55.88 mg·kg^−1^, corresponding to a dissipation efficiency of 69.58%. The addition of either CA or N led to reductions in soil Phe residues to different degrees. Notably, the low-concentration applications of CA and N, whether applied singly or in combination, consistently resulted in lower residual Phe levels compared with their high-concentration counterparts. Among all treatments, the Phy-C1N1 combination achieved the lowest residual concentration (43.06 mg·kg^−1^), with a dissipation rate of 76.56%.

### 3.4. Soil pH, EC and Available Nutrients

**Figure 4:**
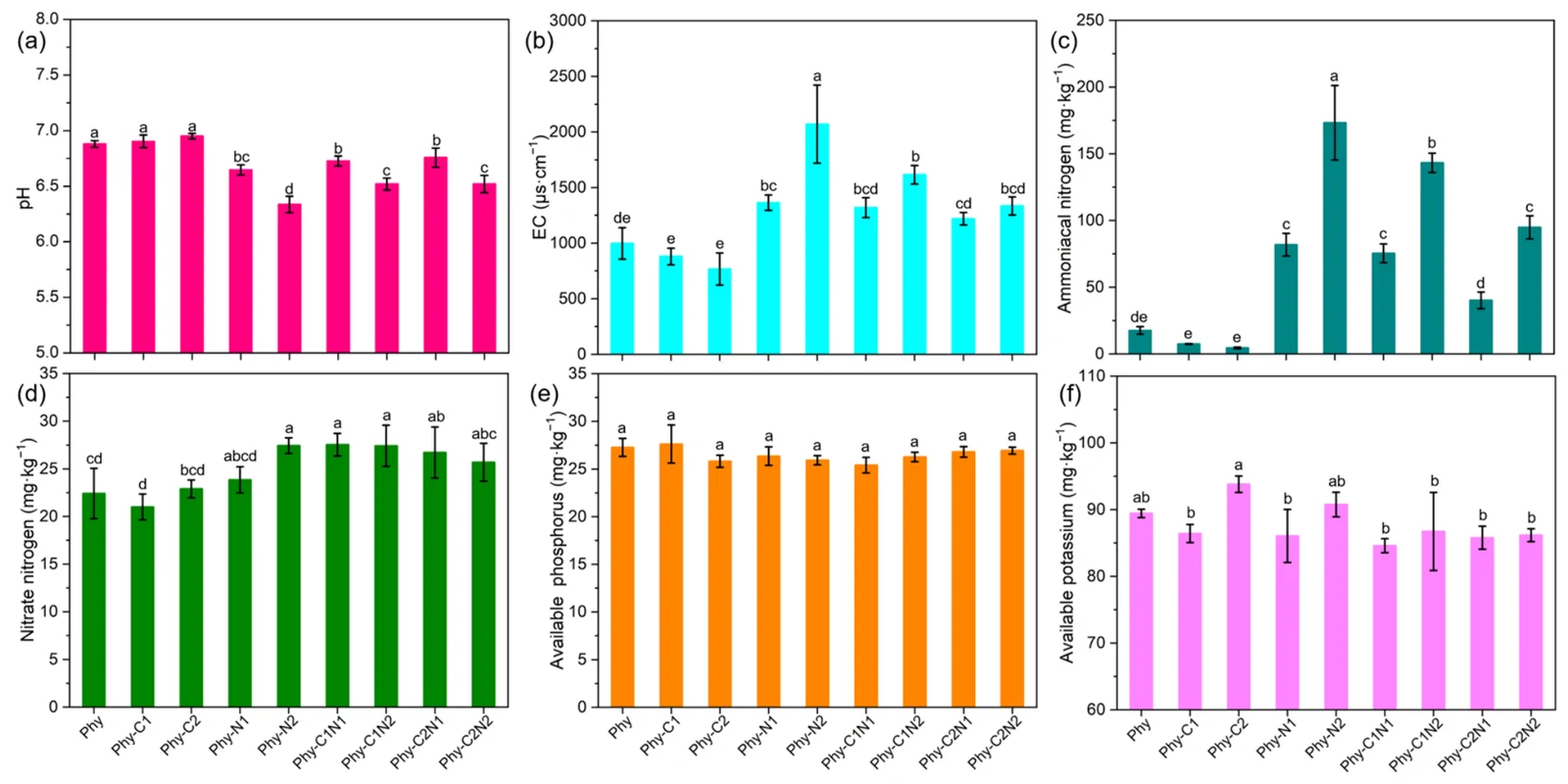
Effects of CA and N levels on the (**a**) pH, (**b**) EC, (**c**) ammonium nitrogen, (**d**) nitrate nitrogen, (**e**) available phosphorus and (**f**) available potassium in the soil. Different lowercase letters mean remarkable variations in various processes (*p* < 0.05).

The application of different chemical additives induced distinct changes in soil physicochemical properties (Figure 4). Soil pH was not significantly affected by the addition of CA alone, whereas N application resulted in a significant pH reduction, with the magnitude of decrease being positively related to the N dosage. In terms of EC, CA addition caused a slight decline, while N addition led to a marked increase in EC values, and higher N concentrations corresponded to greater EC elevations. A similar response pattern was observed for soil NH_4_^+^_N content, where CA addition marginally decreased NH_4_^+^_N levels, whereas N supplementation significantly enhanced them in a dose-dependent manner. For NO_3_^−^_N, no significant changes were detected under CA alone or low-dosage N treatments. However, the application of high-dosage N, as well as the Phy-C1N1, Phy-C1N2, and Phy-C2N1 combinations, resulted in substantial increases in soil NO_3_^−^_N content. AP content remained largely unaffected across all amendment treatments. Similarly, AK levels showed no statistically significant differences from the control (Phy) in most treatments, with the exception that high-dosage CA application significantly elevated AK content compared with its low-dosage counterpart.

### 3.5. Influence of Environmental Factors on Cd Accumulation and Phe Dissipation

**Figure 5:**
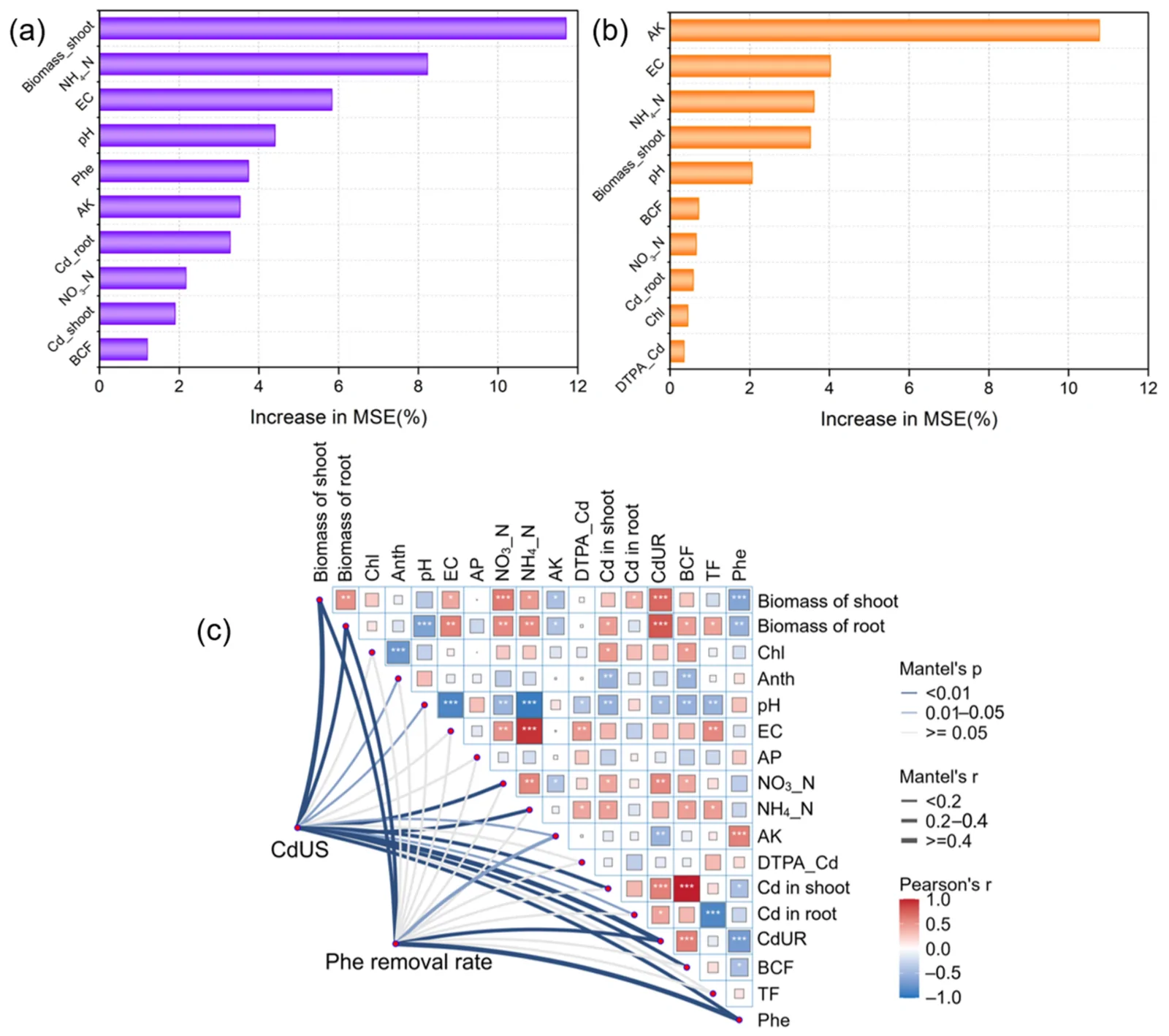
The effects of soil environmental factors and plant factors on the Cd accumulation and Phe dissipation. (**a**,**b**) are the importance ranking diagrams of soil environmental factors and plant factors affecting Cd accumulation (**a**) and Phe dissipation (**b**). (**c**) is the correlations of soil and plant factors with Cd accumulation and Phe dissipation. The edge width corresponds to Mantel’s r value and the edge color denotes statistical significance. Pairwise correlations of soil and plant factors are shown with a color gradient denoting Pearson’s correlation coefficient. * *p* ≤ 0.05, ** *p* ≤ 0.01, *** *p* ≤ 0.001. DTPA_Cd represents the concentration of bioavailable Cd in soil. BCF refers to the bioconcentration factors of Cd; TF refers to the translocation factors of Cd. CdUS indicates the Cd uptake in plant shoots.

**Figure 6:**
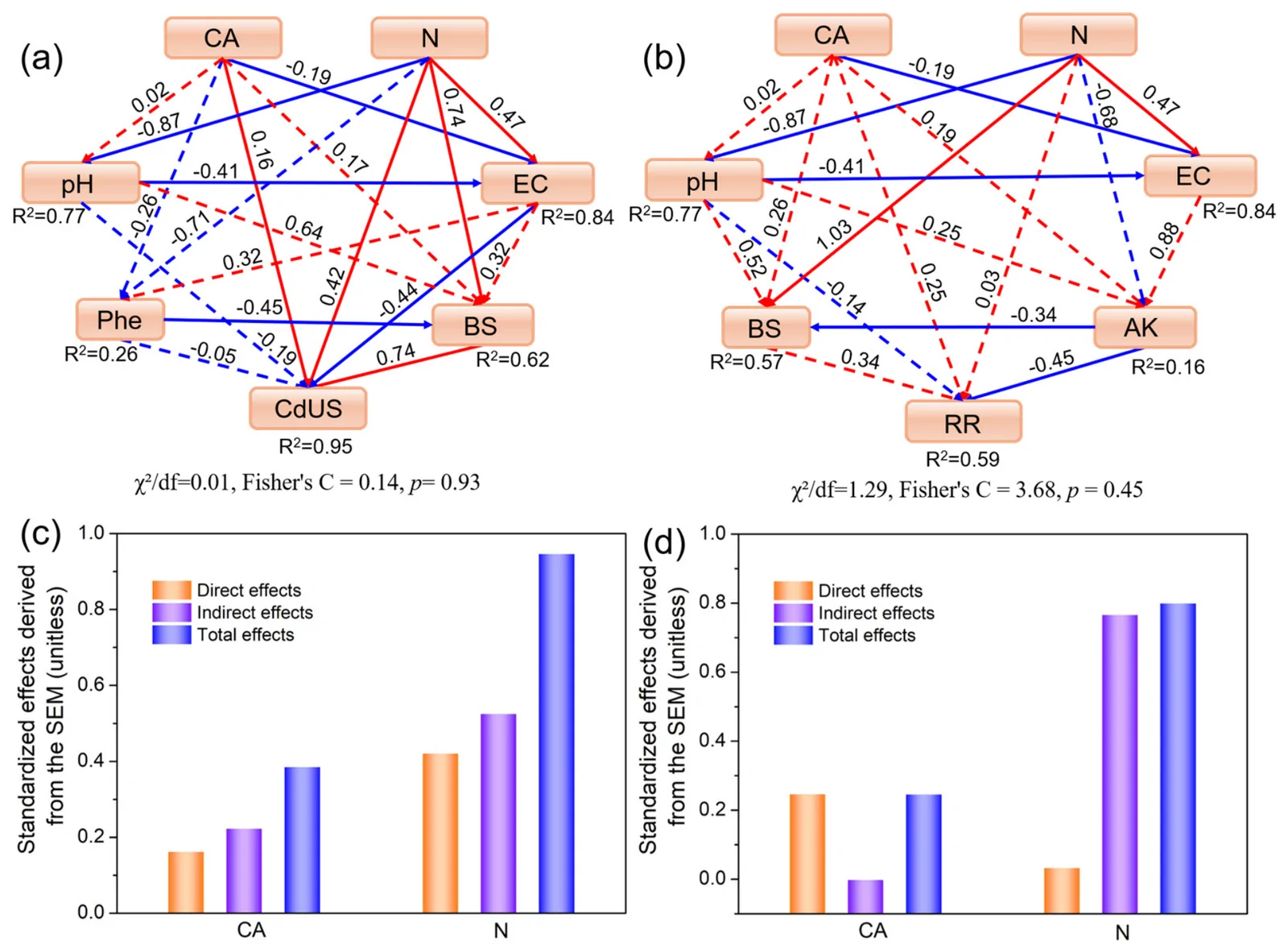
SEM of the effects of soil and plant factors on the Cd accumulation in Marigold (**a**) and Phe dissipation in soil (**b**). The direct and indirect effects of environmental factors and plant factors on Cd accumulation (**c**) and Phe dissipation (**d**). Red and blue arrows correspond to positive and negative relationships, respectively. The solid line and short dashed line indicate significant (*p* < 0.05) and non-significant (*p* > 0.05) relationships, respectively. Numbers adjacent to arrows are standardized path coefficients, analogous to partial regression weights and indicative of the effect size of the relationship. R2 denotes the proportion of variance explained and appears below every response variable in the model. BS represents the plant shoot biomass. CdUS represents the uptake amount of Cd in shoots. RR represents the dissipation rate of Phe.

To elucidate the underlying drivers influencing Cd accumulation in marigold and Phe dissipation in soil following CA and N amendments, we employed a multi-analytical approach combining RF analysis, Mantel tests, and SEM. These analyses examined the relative importance of soil and plant variables, as well as their interconnections with Cd uptake and Phe dissipation (Figure 5 and Figure 6). The RF analysis suggested that shoot biomass was the strongest predictor of Cd accumulation, followed by soil NH_4_^+^_N and EC (Figure 5a). For soil Phe dissipation, AK emerged as the most important determinant, with EC and NH_4_^+^_N also ranking among the top predictors (Figure 5b). Given the exploratory nature of the RF analysis (Table S2), these importance rankings should be interpreted as hypothesis-generating. Mantel tests (Figure 5c) revealed that shoot Cd uptake (CdUS) was significantly and positively correlated with plant biomass, shoot Cd concentration, soil NO_3_^−^_N and NH_4_^+^_N, root Cd uptake, and BCF. Conversely, significant negative correlations were observed between shoot Cd uptake and soil pH, leaf Anth content, and residual Phe concentration. Soil Phe dissipation efficiency showed significant positive associations with marigold biomass, soil AK concentration, and root Cd uptake (CdUR).

The SEM results (Figure 6) further revealed potential direct and indirect pathways through which CA and N exerted their effects. For shoot Cd uptake, the addition of CA (β = 0.16) and N (β = 0.42) both exhibited significant direct positive effects. Indirectly, CA facilitated shoot Cd uptake (total indirect effect = 0.22) through reducing soil EC, decreasing residual Phe concentration, and promoting plant growth, yielding a total effect of 0.39. Nitrogen addition also indirectly enhanced shoot Cd uptake through multiple pathways, including promoting plant growth, lowering soil pH, and reducing residual Phe concentration. However, it also exerted a suppressive effect by elevating soil EC, which partially offset the positive contributions. Collectively, these opposing pathways resulted in a total indirect effect of 0.53 and a total effect of 0.95 for N addition. Regarding Phe dissipation efficiency, both CA (β = 0.25) and N (β = 0.03) exerted positive direct effects. The indirect effect of CA on Phe dissipation was mediated through contrasting pathways: it promoted dissipation by stimulating plant growth, while simultaneously suppressing dissipation via increasing soil pH and AK concentration, resulting in a negligible net indirect effect (−0.001). In contrast, N application indirectly facilitated Phe dissipation (total indirect effect = 0.77) through promoting plant biomass, reducing soil pH, and decreasing AK concentration, all of which collectively contributed to enhanced dissipation efficiency.

## 4. Discussion

### 4.1. Combined Promotion of Marigold Growth by CA and N

The marked disparity in plant growth responses between N and CA treatments underscores their fundamentally distinct physiological roles in modulating marigold performance under Cd–Phe co-contamination. Nitrogen application consistently and significantly enhanced both shoot and root biomass across all N-containing treatments, with the low-dose combination (Phy-C1N1) achieving maximum values (Figure 1a). In stark contrast, CA applied alone produced no statistically significant changes in biomass. This divergence stems from their fundamentally distinct modes of action. As an essential macronutrient, N directly participates in chlorophyll biosynthesis, protein synthesis, and overall carbon assimilation [13], thereby alleviating the Cd-induced nitrogen deficiency that commonly constrains photosynthetic capacity and vegetative growth under heavy metal stress [25]. The SEM results (Figure 6) quantitatively substantiate this interpretation, revealing a substantial direct positive effect of N on shoot biomass, which constitutes the primary pathway through which N enhances phytoremediation potential. By contrast, CA functions predominantly as a metal-chelating agent rather than a nutrient source [26]. Although CA can be metabolized by rhizosphere microorganisms, its contribution as a direct carbon source for plant growth within the short-term greenhouse experimental period is negligible compared to the profound nutritional effect of N [26,27]. Consequently, the absence of growth promotion under CA-alone treatment is biologically expected.

The inverse Chl–Anth relationship observed in this study is physiologically informative. Anth are stress-induced secondary metabolites that accumulate as protective antioxidants under abiotic stresses [28], while Chl content directly reflects photosynthetic capacity. The concurrent Chl elevation and Anth decline therefore signals effective alleviation of Cd–Phe co-stress, wherein the diminished requirement for defensive pigmentation indicates restored photosynthetic function [29]. Previous investigations have established that the recovery of photosynthetic pigments is closely associated with the mitigation of oxidative damage and the preservation of chloroplast integrity under heavy metal stress [12]. Extending this line of evidence, the present study reveals that N supplementation exerts a pronounced effect on pigment recovery [30]. The superior Chl response and Anth reduction under high-N treatments suggest that N not only directly fuels Chl biosynthesis but also potentiates the plant’s intrinsic antioxidative defense capacity, effectively scavenging reactive oxygen species generated by co-contaminant stress. Previous studies have shown that N can enhance stress tolerance by maintaining redox homeostasis and preserving chloroplast ultrastructure [31], which may partly explain the observed pigment recovery in our N-treated plants. Collectively, these findings establish a clear physiological division of labor between N and CA in supporting marigold performance under co-contamination: N drives biomass accumulation through nutritional replenishment, while CA, though ineffective alone, complements this process by mitigating Cd-induced oxidative stress. This physiological synergy, particularly at the low-dose combination (Phy-C1N1), provides the material foundation for subsequent enhancement of phytoremediation performance.

### 4.2. Combined Rhizosphere Chemical Effects of CA and N in Enhancing Cd Phytoextraction

Enhanced Cd accumulation in marigold shoots under combined CA and N amendments can be attributed to their distinct yet complementary rhizosphere chemical actions. Nitrogen fertilization plays a multifaceted role in promoting Cd phytoextraction. First and foremost, N serves as an essential macronutrient that directly enhances biomass production, creating a larger sink capacity for Cd accumulation in aboveground tissues [32]. Beyond this nutritional effect, ammonium-based N fertilizers acidify the rhizosphere through proton release during NH_4_^+^ assimilation, which increases Cd solubility and elevates DTPA-extractable Cd concentrations, thereby enhancing Cd bioavailability for root uptake [13]. Additionally, N modulates physiological and metabolic processes in plant cells that facilitate Cd uptake and translocation [33]. Collectively, these mechanisms establish N as a potent enhancer of Cd phytoextraction.

However, the influence of N on Cd phytoextraction is not unidirectional. While N fertilization enhances Cd solubility and promotes plant growth, it simultaneously elevates soil EC through the accumulation of soluble salts. This N-induced EC surge creates osmotic stress that restricts Cd uptake from soil to roots, impairs root water uptake and diminishes transpirational pull [34]—the primary driving force for Cd transport from roots to shoots via xylem [35]. The elevated EC under high N likely restricted Cd translocation to aboveground tissues, limiting overall phytoextraction efficiency. Critically, CA exerted a buffering effect by reducing the N-induced EC surge (Figure 2 and Figure 6a), thereby maintaining a more favorable ionic environment that preserved root hydraulic conductivity and xylem loading capacity. This CA-mediated EC moderation ensured that Cd mobilized into the soil solution could be efficiently transported to the shoots, sustaining phytoextraction performance even under N fertilization.

Beyond its role in EC buffering, CA contributes to Cd phytoextraction through additional mechanisms. CA is widely recognized as an effective chelating agent for enhancing phytoremediation, owing to its ability to increase the solubility and bioavailability of heavy metals in soil through ligand-promoted dissolution and complexation reactions [8,12]. In the present study, CA amendment indeed significantly increased Cd concentrations in marigold shoots (Figure 2a), confirming its role in facilitating Cd transfer from soil to plants. However, contrary to the expectation that CA would elevate DTPA-Cd in soil, no significant increase was observed under CA treatments at harvest. This apparent discrepancy is not without precedent. A recent study on purslane (*Portulaca oleracea*) similarly reported that Cd extracted with DTPA remained unchanged in citric acid treatments compared to the control, despite significant increases in Cd concentrations in aboveground plant tissues [36]. CA rapidly enhances Cd solubility upon application, but the mobilized Cd is continuously absorbed by plant roots and depleted from the soil solution over the course of the experiment [8]. Consequently, the residual DTPA-Cd pool measured at harvest represents only the remaining bioavailable fraction after sustained root uptake, rather than the initial solubilization flux. The elevated shoot Cd concentrations thus provide evidence that CA effectively facilitated Cd mobilization and plant uptake, even though the final DTPA-Cd concentration did not differ from the control. The combined regulation of rhizosphere chemistry and plant physiology by CA and N, rather than either amendment alone, optimized Cd phytoextraction performance. Among the tested treatments, Phy-C1N1 achieved the highest shoot Cd uptake and Phe dissipation rate while using relatively lower amendment inputs.

### 4.3. Combined Effects of CA and N on Phe Dissipation

The dissipation of Phe in the co-contaminated soil exhibited a distinct response pattern to CA and N amendments. Compared with the marigold-only control (Phy), all treatments containing CA or N enhanced Phe dissipation to varying degrees, with the low-dose combination (Phy-C1N1) achieving the highest dissipation rate (Figure 3). Our findings are consistent with previous reports demonstrating that both LMWOAs and nitrogen fertilization can significantly improve the efficiency of phytoremediation for PAH-contaminated soils [14,17]. The enhanced Phe dissipation observed in the present study can be attributed to several complementary mechanisms. First, it is reasonable to infer that CA may have facilitated Phe desorption from soil particles by disrupting cation bridges, as previously demonstrated in related studies [14,26]. Second, based on previous findings, N supplementation alleviated nutrient limitation in the rhizosphere, stimulating the metabolic activity and proliferation of indigenous microbial communities capable of PHE dissipation. Nitrogen application has been shown to improve the abundance and diversity of soil microbial communities and enhance the expression of functional genes involved in PAH detoxification [16]. Collectively, these findings indicate that the combination of CA and N enhances Phe dissipation through a synergistic interplay of increased contaminant bioavailability and stimulated microbial metabolic activity. This integrated chemical–biological mechanism underscores the potential of combined amendment strategies for improving the phytoremediation of organic pollutants in co-contaminated soils.

While the SEM revealed several statistically significant pathways consistent with our conceptual framework, and cross-validation and bootstrap stability assessment indicated stable model performance for the random forest analysis, both multivariate analyses were based on a relatively small sample size (*n* = 27). Therefore, the results of these analyses should be interpreted as exploratory and hypothesis-generating rather than confirmatory. Future studies with larger sample sizes and independent validation datasets are necessary to confirm the robustness of these relationships. In addition, while the observed decrease in residual Phe concentration was described as dissipation, the specific contributions of biodegradation, plant uptake, volatilization, and sorption to soil organic matter were not distinguished. Future investigations incorporating metagenomic, transcriptomic, and metabolomic analyses, as well as detailed metabolite profiling of Phe degradation products, will be needed to elucidate the underlying dissipation mechanisms and achieve a complete mass balance. Finally, the harvested Cd-enriched plant biomass should be properly collected and disposed of as hazardous waste to prevent secondary contamination, rather than being returned to the soil or discarded in the environment.

## 5. Conclusions

This study demonstrated that the combined application of CA and N significantly enhances phytoremediation performance in Cd–Phe co-contaminated soil, yet their combined action operates through distinct and complementary pathways that are dose-dependent. N serves as the primary driver of plant biomass production by directly providing essential nutrients and promoting photosynthetic pigment synthesis, whereas CA alone does not confer direct growth benefits within the experimental timeframe. For Cd phytoextraction, N promotes Cd uptake through rhizosphere acidification and enhanced biomass production, but excessive N inputs elevate soil EC, imposing osmotic stress that restricts Cd uptake from soil to roots. CA mitigates this constraint by buffering the N-induced EC surge. This EC-moderation effect, together with CA’s metal-chelating function, ensures that mobilized Cd is efficiently transported to aboveground tissues. Regarding Phe dissipation, the combined application of CA and N influences rhizosphere nutrient availability and plant growth, which in turn promote Phe dissipation. Among the tested treatments, the low-dose combination (Phy-C1N1) exhibited the most favorable balance of remediation performance and amendment input, and is therefore recommended for further field validation. These findings advance the mechanistic understanding of CA-N synergy in phytoremediation systems and offer a new perspective for optimizing combined amendment strategies in co-contaminated environments.

## Figures and Tables

**Table 1:** Experimental design of marigold with different additives.

Treatment	Description
Phy	Marigold alone
Phy-C1	Marigold couple with 1 g citric acid per pot
Phy-C2	Marigold couple with 2 g citric acid per pot
Phy-N1	Marigold couple with 0.1 g N (0.286 g NH_4_NO_3_) per pot
Phy-N2	Marigold couple with 0.2 g N (0.572 g NH_4_NO_3_) per pot
Phy-C1N1	Marigold couple with 1 g citric acid and 0.1 g N (0.286 g NH_4_NO_3_) per pot
Phy-C1N2	Marigold couple with 1 g citric acid and 0.2 g N (0.572 g NH_4_NO_3_) per pot
Phy-C2N1	Marigold couple with 2 g citric acid and 0.1 g N (0.286 g NH_4_NO_3_) per pot
Phy-C2N2	Marigold couple with 2 g citric acid and 0.2 g N (0.572 g NH_4_NO_3_) per pot

## Data Availability

Dataset available on request from the authors.

## Supplementary Materials

The following supporting information can be downloaded at https://www.mdpi.com/article/10.3390/toxics14090770/s1. Table S1: The detailed application concentrations and volumes of additives; Table S2: The full random forest parameters; Table S3: Two-way ANOVA results for main response variables.
