# Supplementary material for: The Combined Effects of Citric Acid and Ammonium Nitrate on the Phytoremediation of Cd-Phenanthrene Co-Contaminated Soil Using Marigold
Source: Toxics. 2026 Aug 28;14(9):770. doi: 10.3390/toxics14090770 (PMC13611540; doi:10.3390/toxics14090770)
Supplement: Supplementary file 1 [file toxics-14-00770-s001.zip › toxics-4509217-supplementary.pdf]

Supplementary Materials to "The Combined Effects of Citric Acid and Ammonium Nitrate on the Phytoremediation of Cd-Phenanthrene Co-Contaminated Soil Using Marigold"

Xinzhuo Qian [1,†], Ting Xu [2,3,†], Xuemei Zhong [1,*], Weijin Zheng [4], Lizhu Yuan [4,*] and Bo Song [2]

[1] College of Earth Sciences, Guilin University of Technology, Guilin 541004, China
[2] College of Environmental Science and Engineering, Guilin University of Technology, Guilin 541004, China
[3] Guangxi Kangwanjia Farmland Soil Remediation Co., Nanning 530006, China
[4] Research Center for Eco-Environmental Engineering, Dongguan University of Technology, Dongguan 523808, China
[*] Correspondence: zxm@glut.edu.cn (X.Z.); 2022019@dgut.edu.cn (L.Y.)
[†] These authors contributed equally to this work.

Table S1. The detailed application concentrations and volumes of additives.

Treatment	Concentrations ($g·L^{-1}$)	Volume (ml)
Phy	0 $g·L^{-1}$	0
Phy-C1	10 $g·L^{-1}$ CA	33.3
Phy-C2	10 $g·L^{-1}$ CA	66.7
Phy-N1	1 $g·L^{-1}$ N	33.3
Phy-N2	1 $g·L^{-1}$ N	66.7
Phy-C1N1	10 $g·L^{-1}$CA and 1 $g·L^{-1}$ N	33.3
Phy-C1N2	5 $g·L^{-1}$CA and 1 $g·L^{-1}$ N	66.7
Phy-C2N1	10 $g·L^{-1}$CA and 0.5 $g·L^{-1}$ N	66.7
Phy-C2N2	10 $g·L^{-1}$CA and 1 $g·L^{-1}$ N	66.7

Table S2. The full random forest parameters.

Parameter	Cd	Phe
Number of trees (ntree)	500	500
Variables per split (mtry)	5	5
Number of predictors (p)	16	15
OOB MSE	28.422	4.293
OOB RMSE	5.331	2.072
OOB relative error (%)	19.650	2.810
OOB R^2	0.486	0.252
CV MSE (mean ± SD)	26.434± 8.798	4.101± 1.440
CV R^2 (mean ± SD)	0.471±0.119	0.216± 0.078

Table S3. Two-way ANOVA results for main response variables.

Response variable	Source	df	F	p
Biomass of shoot	CA	0.060	3.087	0.070
	N	0.398	20.595	0.000
	CA×N	0.007	0.371	0.826
Biomass of root	CA	0.000	0.153	0.859
	N	0.002	27.153	0.000
	CA×N	0.000	1.717	0.190
Cd concentration in shoot	CA	18.818	5.042	0.018
	N	46.345	12.417	0.000
	CA×N	2.632	0.705	0.599
Cd concentration in root	CA	31.929	17.254	0.000
	N	1.003	0.542	0.591
	CA×N	3.649	1.972	0.142
Cd uptake amounts in shoot	CA	137.217	14.891	0.000
	N	484.298	52.556	0.000
	CA×N	20.796	2.257	0.103
Cd uptake amounts in root	CA	0.071	17.554	0.000
	N	0.290	71.378	0.000
	CA×N	0.010	2.418	0.086
Phe removal rate	CA	14.534	5.955	0.010
	N	32.321	13.244	0.000
	CA×N	4.306	1.764	0.180
